# Rutaecarpine Ameliorates Ethanol-Induced Gastric Mucosal Injury in Mice by Modulating Genes Related to Inflammation, Oxidative Stress and Apoptosis

**DOI:** 10.3389/fphar.2020.600295

**Published:** 2020-11-26

**Authors:** Sichen Ren, Ying Wei, Ruilin Wang, Shizhang Wei, Jianxia Wen, Tao Yang, Xing Chen, Shihua Wu, Manyi Jing, Haotian Li, Min Wang, Yanling Zhao

**Affiliations:** ^1^ School of Pharmacy, Chengdu University of Traditional Chinese Medicine, Chengdu, China; ^2^ Department of Pharmacy, The Fifth Medical Center of Chinese PLA General Hospital, Beijing, China; ^3^ Integrative Medical Center, The Fifth Medical Center of Chinese PLA General Hospital, Beijing, China; ^4^ School of Clinical Medicine, Chengdu University of Traditional Chinese Medicine, Chengdu, China

**Keywords:** rutaecarpine, ethanol, gastric mucosal injury, anti-inflammation, anti-oxidation, anti-apoptosis

## Abstract

**Background:** Rutaecarpine (RUT), a major quinazolino carboline alkaloid compound from the dry unripe fruit *Tetradium ruticarpum* (A. Juss.) T. G. Hartley, has various pharmacological effects. The aim of this present study was to investigate the potential gastroprotective effect of rutaecarpine on ethanol-induced acute gastric mucosal injury in mice and associated molecular mechanisms, such as activating Nrf2 and Bcl-2 via PI3K/AKT signaling pathway and inhibiting NF-κB.

**Methods:** Gastric ulcer index and histopathology was carried out to determine the efficacy of RUT in gastric ulceration, and the content of SOD, GSH in serum and CAT, MDA, MPO, TNF-α, IL-6, IL-1β in tissue were measured by kits. Besides, in order to illustrate the potential inflammatory, oxidative, and apoptotic perturbations, the mRNA levels of NF-κB p65, PI3K, AKT, Nrf2, Nqo1, HO-1, Bcl-2 and Bax were analyzed. In addition, the protein expression of NF-κB p65 and Nrf2 in cytoplasm and nucleus, AKT, p-AKT, Bcl-2 Bax and Caspase 3 were analyzed for further verification. Finally, immunofluorescence analysis was performed to further verify nuclear translocation of NF-κB p65.

**Results:** Current data strongly demonstrated that RUT alleviated the gross gastric damage, ulcer index and the histopathology damage caused by ethanol. RUT inhibited the expression and nuclear translocation of NF-κB p65 and the expression of its downstream signals, such as TNF-α, IL-6, IL-1β and MPO. Immunofluorescence analysis also verifies the result. In the context of oxidative stress, RUT improved the antioxidant milieu by remarkably upregulating the expression Nqo1 and HO-1 with activating Nrf2, and could remarkably upregulate antioxidant SOD, GSH, CAT and downregulate levels of MDA. Additionally, RUT activate the expression of Bcl-2 and inhibited the expression of downstream signals Bax and Caspase 3 to promote gastric cellular survival. These were confirmed by RUT activation of the PI3K/AKT pathway manifested by enhanced expression of PI3K and promotion of AKT phosphorylation.

**Conclusion:** Taken together, these results strongly demonstrated that RUT exerted a gastroprotective effect against gastric mucosal injury induced by ethanol. The underlying mechanism might be associated with the improvement of anti-inflammatory, anti-oxidation and anti-apoptosis system.

## Introduction

Gastric ulcer (GU) is a common multifactorial gastrointestinal disease worldwide, affecting the quality of life of millions of patients. According to the survey, 20–60 people out of every 100,000 population suffer from GUs, accounting for 5–10% of the world's mortality (Sung et al., 2010). Under normal physiological conditions, the mucosa maintains its integrity through defense, thus maintaining the gastric epithelial barrier and blood flow, as well as the presence of protective factors such as mucus, prostaglandins, bicarbonate, heat shock protein (HSP) and growth factors. Mucosal damage may occur when the toxic factors such as gastric acid, pepsin, bile acid, ethanol, *Helicobacter pylori* and nonsteroidal anti-inflammatory drugs (NSAIDs) invade and exceed the defense function load of gastric mucosa (Guth et al., 1979; Lee et al., 2017; Khan et al., 2018). Some other factors, such as inadequate dietary habits, excessive ethanol consumption, cigarette smoking, stress and hereditary predisposition, also may lead to the development of GU (Søreide et al., 2015; Jeon et al., 2020). Yet, the clinical treatment for the disease focuses on the use of antisecretory drugs such as H2 receptor antagonist (cimetidine) or proton pump inhibitors (omeprazole), antibiotics (clarithromycin), antacids (aluminum hydroxide), prostaglandin analogues and mucosal protective agents (bismuth) currently (Wallace, 2008). Although the use of these drug is associated with the problem of reoccurrence of GU and several undesirable side effects, the usage is still on the rise, which brings more risks (Piao et al., 2018). Therefore, the search for alternative drugs or natural resources has attracted many scholars.

**FIGURE 1:**
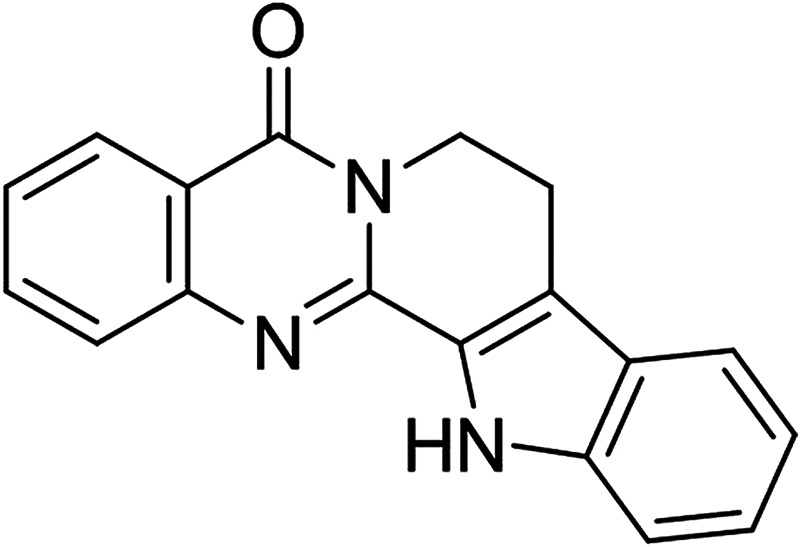
The chemical structure of rutaecarpine.

With the deepening of the research on natural drugs, the characteristics of some natural drugs with rich sources and less side effects have been recognized. Rutaecarpine (RUT, Figure 1), a major quinazolino carboline alkaloid compound from the dry unripe fruit (named “Wu-Chu-Yu” in China) of *Tetradium ruticarpum* (A. Juss.) T. G. Hartley. It is a longstanding and multipurpose Chinese medicine traditionally used for the treatment of abdominal pain, vomiting and pyresis (Zhao et al., 2015). As one of the main active components of “Wu-Chu-Yu”, RUT has a wide range of biological and pharmacological effects, such as diuresis, perspiration, uterotonic action, cardiovascular protection, improving brain function, protecting gastric mucosa, anti-inflammatory and anti-oxidation, and its pharmacological mechanism involves a variety of biological targets (Jia et al., 2010; Tian et al., 2019). Several *in vitro* studies acknowledged positive effect of RUT in peritoneal resident macrophages (Li et al., 2019), monocytes (Liu et al., 2016), osteoclast (Fukuma et al., 2018), HepG2 cells (Jin et al., 2017; Surbala et al., 2020) and Hepa-1c1c7 cells (Lee et al., 2012). Meanwhile, RUT has shown versatile beneficial effects on several experimental models, such as colitis (Zhang et al., 2020), atherosclerosis (Luo et al., 2020), cerebral ischemia-reperfusion (Han et al., 2019), hypertension (Ma et al., 2019), Acute kidney injury (Liu et al., 2020a; Liu et al., 2020b), type 2 diabetic (Surbala et al., 2020), Alzheimer’s disease (Ahmad et al., 2020; Huang et al., 2020) and so on. Notably, RUT exerted definite anti-inflammatory, anti-oxidation and anti-apoptosis effects in several experimental pathology with the signaling of NF-κB, Nrf2/HO-1, Bcl-2/Bax pathways (Jin et al., 2017; Li et al., 2019a; Li et al., 2019b; Han et al., 2019). Yet, few reports focused on the effect of RUT on the ethanol-induced gastric ulcer based on above pathways. Hence, the aim of the current study was to investigate the ameliorative activity of RUT on ethanol-induced gastric ulcer and explore its underlying mechanisms.

## Materials and Methods

### Reagents

In this study, RUT (pure: ≥98%) were purchased from Chengdu Chroma-Biotechnology Company (Chengdu, China). As the positive control, omeprazole (OME) was supplied by AstraZeneca Research-based Biopharmaceutical Company (Sweden). Biochemical indicator kits for SOD (Cat.No.: A001-3-2), GSH (Cat.No.: A006-2-1), CAT (Cat.No.: A007-1-1), MDA (Cat.No.: A003-1-1) and MPO (Cat.No.: A044-1-1) were provided by the Nanjing Jiancheng Bioengineering Institute (Nanjing, China) and enzyme-linked immune sorbent assay (ELISA) kits for TNF-α (Cat.No.: ml002293), IL-6 (Cat.No.: ml002293) and IL-1β (Cat.No.: ml063132) were provided by Shanghai enzyme linked biology Co., Ltd. (Shanghai, China). All antibodies were provided by Cell Signaling Technology, Inc. (Danvers, MA, United States), Abcam plc. (Cambridge, United Kingdom) or Proteintech Group, Inc. (Rosemont, IL, USA), and the specific information is shown in Table 1. All other experimental supplies were purchased from commercial sources.

**TABLE 1:** Antibodies information.

Antibodies	Dilution	Manufacturers	Cat. No.
For western blot analysis			
Rabbit anti-NF-κB p65	1:1,000	Cell signaling technology	8242T
Rabbit Anti-Nrf2	1:1,000	Proteintech	16396-1-AP
Rabbit anti-Bcl-2	1:1,000	Cell signaling technology	3498T
Rabbit anti-Bax	1:1,000	Cell signaling technology	2772T
Rabbit anti-cleaved Caspase-3	1:5,000	Abcam	ab214430
Rabbit Anti-AKT	1:1,000	Proteintech	10176-2-AP
Mouse Anti-pAKT	1:5,000	Proteintech	66444-1-lg
Rabbit Anti-GAPDH	1:10000	Proteintech	10494-1-AP
Rabbit anti-histone H3	1:2000	Cell signaling technology	4499T
For immunofluorescence analysis			
Rabbit anti-NF-κB p65	1:500	Cell signaling technology	8242T

### Animals and Treatments

A total of 50 male specific pathogen-free (SPF) KM mice weighting 20 ± 2 g were obtained from SPF (Beijing) Biotechnology Co., Ltd. (Permission No. SCXK-(A) 2012-0004). All the animals were maintained in the same temperature (25 ± 2°C) and lighting (12:12 h light:dark cycle) conditions for 1 w and provided with water and standard chow ad libitum. All experimental procedures were approved by the Animal Experiment Committee of Fifth Medical Centre, General Hospital of Chinese People’s Liberation Army, and carried out in accordance with the guidelines of the Council on Animal Care of Academia Sinica.

All the animals were randomly assigned five groups with ten mice in each group. RUT and OME were prepared as a stock solution in 0.5% carboxymethyl cellulose sodium (CMC-Na) and diluted to the appropriate dosage or concentration. In the first 3 days, RUT of 450 and 900 μg/kg were administered intragastrically to RUT low dose group and RUT high dose group, respectively. OME group were administered omeprazole 20 mg/kg, while normal control group and model group were given the vehicle (0.5% CMC-Na). On the third day, 2 h after each group administration, normal control group received normal saline while other groups intragastrically received ethanol (10 ml/kg) instead. The animals were deprived of food for 24 h before the experiments but allowed free access to water and placed in wire cages to avoid interference from litter and feces. Finally, the animals were sacrificed after 2 h and their serum and gastric tissues were harvested for the further studies.

### Macroscopic Assessment of Mucosal Lesions

Stomachs were removed and expanded along the larger curvature and rinsed thoroughly in saline solution. Then two observers who unaware of the treatment measured the lesion size with a vernier caliper and a magnifying glass, and calculated the ulcer index (UI) according to the Guth standard (Guth et al., 1979): no lesion (score 0), epithelial lesion or the lesion <1 mm (score 1), 1 mm ≤ lesion < 2 mm (score 2), 2 mm ≤ lesion <3 mm (score 3), 3 mm ≤ lesion <4 mm (score 4), 4 mm ≤ lesion (score segmentally), and twice for width > 1 mm. The scores were relative values, and the average UI of each group was obtained by dividing the total scores by the number of animals. The inhibition effect (%) of each protective material was calculated by using the following formula:

Ulcer inhibition%=(UI in model−UI in test)×100/UI in model

### Histological Analysis

Gastric tissues were excised and fixed in 10% buffered formalin for more than 48 h. After dehydrating in gradient alcohol and embedding in paraffin, three or four paraffin-embedded sections (4–5 μm thick) were prepared and stained with hematoxylin and eosin (H&E) for histological evaluation. Then the pathological changes in the gastric tissues were observed under a Nikon microscope (Nikon Instruments Inc., Japan) and analyzed by NIS-Elements (version F 4.0, Japan) software.

### Determinations of GSH, SOD, CAT, MDA, MPO, TNF-α, IL-6, IL-1β Levels

Blood samples were collected and centrifuged at 3,000 rpm for 10 min, and then the serum samples were stored at −80°C until analysis. Gastric tissues were homogenized with cold saline and centrifuged at 12,000 rpm at 4°C for 10 min, and the supernatant of the homogenate was collected and stored at −80°C. The serum levels of SOD, GSH and the tissue levels of CAT, MDA, MPO, TNF-α, IL-6, IL-1β were measured (Liu et al., 2010; Yang et al., 2019). In brief, according to the manufacturer’s protocols, the level of SOD were tested by water-soluble tetrazolium-1 (WST-1) method, the final color was read at 450 nm. Then we used ammonium molybdate method to measure the change of CAT at 405 nm and the protein level was detected by BCA Protein Assay kit (Beijing Solarbio Science & Technology Co., Ltd. Beijing, China), because ammonium molybdate can stop decomposition of H_2_O_2_ by CAT and form a yellow complex with remaining H_2_O_2_. Based on the principle that GSH can react with dithiodinitrobenzoic acid (DTNB), we quantitatively determined the content of GSH in serum by colorimetry at 405 nm. Additionally, the MDA activity were detected by thiobarbituric acid (TBA) method, the final color was detected at 450, 532, and 600 nm. According to the capability of MPO contained in neutrophils to reduce the hydrogen peroxide, we quantitatively detected the content of MPO at 460 nm to determine the number of neutrophils. For TNF-α, IL-6, IL-1β, we used enzyme-linked immune sorbent assay (ELISA) to implement. The detection operations of all these experiments were carried out on Synergy Hybrid Reader (Biotek, Winooski, VT, United States).

### Quantitative RT-PCR Analyses

The mRNA expressions of NF-κB, PI3K, Nrf2, Nqo1, HO-1, Bcl-2, and Bax in the gastric tissue were detected by quantitative reverse transcription polymerase chain reaction (RT-PCR). According to the manufacturer’s protocol, total RNA was isolated from about 40 mg frozen gastric tissues of each group using Trizol reagent (Life Technologies, CA, United States). The RNA concentration was measured at 260 and 280 nm on a spectrophotometer (Purity (A260/A280) was considered qualified within the threshold of 1.8–2.2). Hereafter, RNA (2 μg) was reverse-transcribed using a RevertAid First Strand cNDA Synthesis Kit (Thermo Fisher Scientific, MA, United States). The program setting was: incubate for 5 min at 25°C followed by 60 min at 42°C and terminate the reaction by heating at 70°C for 5 min, and the obtained cDNA was stored at −20°C for subsequent PCR reactions. At last, 2 μL cDNA and SYBR^™^ Select Master Mix (Applied Biosystem, CA, United States) was used and then PCR amplification was carried out by ABI 7500 Real Time PCR machine (Applied Biosystems Inc., Carlsbad, CA, United States), running 45 cycles at 95°C for 5 s and 60°C for 60 s. The method recommended in Minimum Information for the Publication of Quantitative Real-Time PCR Experiments (MIQE) was strictly followed (Bustin et al., 2009). The data was calculated through 2^−△△CT^ method with β-actin as an endogenous reference. Table 2 listed the primers used in this study.

**TABLE 2:** Primers sequences for RT-PCR.

Gene	Sense primer (5′-3′)	Antisense primer (5′-3′)
NF-κB p65	TCCAGGCTCCTGTTCGAGTCTC	CGGTGGCGATCATCTGTGTCTG
PI3K	CTGAGAACGCCACCGCCTTG	TCCACCACGACTTGACACATTAGC
Nrf2	GTAGATGACCATGAGTCGCTTGCC	CTTGCTCCATGTCCTGCTCTATGC
Nqo1	AGGCTGCTGTAGAGGCTCTGAAG	GCTCAGGCGTCCTTCCTTATATGC
HO-1	ACCGCCTTCCTGCTCAACATTG	CTCTGACGAAGTGACGCCATCTG
Bcl-2	TCCTTCCAGCCTGAGAGCAACC	TCACGACGGTAGCGACGAGAG
Bax	CGTGAGCGGCTGCTTGTCTG	ATGGTGAGCGAGGCGGTGAG
β-actin	ATCACTATTGGCAACGAGCGGTTC	CAGCACTGTGTTGGCATAGAGGTC

### Western Blot Analysis

Mice gastric tissue (50 mg) was homogenized and lysed in ice-cold radio immunoprecipitation assay (RIPA) lysis buffer containing 1% phenylmethylsulfonyl fluoride (PMSF), phosphatase inhibitor and protease inhibitor cocktail. Subsequently, the samples were centrifuged at 10,000 g and 4°C for 10 min. After centrifugation, the supernatant was collected and subjected to BCA Protein Assay kit (Beijing Solarbio Science & Technology Co., Ltd. Beijing, China) to measure protein concentration. In addition, nuclear and cytoplasmic extraction were performed using a nuclear extraction kit (Beijing Solarbio Science & Technology Co., Ltd., Beijing, China) to detect the presence of NF-κB and Nrf2 in nuclear and cytoplasmic proteins. Specifically, according to the manufacturer's instructions, 20 mg gastric tissue homogenate was vortexed at high speed for 15 s in 100 μl cytoplasmic protein extraction reagent containing 1% PMSF and then ice bath for 10 min. Subsequently, the samples were vortexed again for 10 s and centrifuge at 15,000 g at 4°C for 10 min. The resulting supernatant was cytoplasmic protein, whereas the precipitate was nuclear protein. Later, the supernatant was separated completely and 50 ul nuclear protein extraction reagent was added into the nuclear protein. The supernatant containing nucleoprotein was obtained by the same method. Similarly, their protein concentration was determined by BCA Protein Assay kit. The samples were denatured with reducing Laemmli SDS sample buffer and soaked in water at 95°C for 5 min. For Western blot analysis, the same amount of protein from all lysates of each sample was separated by 10–12% sodium dodecyl sulfate-polyacrylamide gel electrophoresis (SDS-PAGE), and then transferred onto the polyvinylidene fluoride (PVDF) membranes. Then immunodetection was carried out using corresponding primary antibodies (Table 1) in a solution of 5% bovine serum albumin (BSA), Tris-buffered saline (TBS), and 0.05% Tween 20 overnight at 4°C after blocked in BSA at room temperature for 2 h. GAPDH was used as the control for total and cytosolic protein extract, while Histone H3 was the control for nuclear protein extract. After incubation with the appropriate secondary antibodies at room temperature for 2 h, the membranes were washed 3 times in TBST (TBS with Tween 20), and the immunoreactive protein was measured using a chemiluminescence system. All Western blot studies were repeated three times.

### Immunofluorescence Analysis

In order to further verify that RUT reversed ethanol-induced NF-κB translocation from the cytoplasm to the nucleus, immunofluorescence analysis was performed. After deparaffinization and dehydration in gradient alcohol, gastric tissue paraffin sections were placed in ethylenediaminetetraacetic acid (EDTA) for antigen retrieval. Then the sections were washed three times with phosphate-buffered saline (PBS) and blocked with 1% BSA for 30 min at room temperature. Subsequently, sections were incubated with anti-NF-κB p65 antibody (1:500, Table 1) in a solution of 5% BSA and PBS at 4°C overnight in a damp box. After washed three times the next day, the sections were incubated with the appropriate secondary antibody for 1 h at room temperature. Later, 4,6-diamidino-2-phenylindole (DAPI) was used to stain nuclei and images were taken with a confocal microscope (NIKON Eclipse C1, Nikon Instruments Inc., Japan) and analyzed by NIKON DS-U3 (Nikon Instruments Inc., Japan). Finally, we use Image-Pro Plus (version 6.0, Media Cybemetics, INC., Rockville, MD, United States) to measure and inregrate the optical density and total per area for a statistical analysis of Immunofluorescence staining.

### Statistical Analysis

The experimental data were expressed as mean ± standard deviation (SD), and analyzed with the SPSS software program (Version 24.0, SPSS Inc., Chicago, IL, United States). Differences between groups were evaluated by one-way analysis of variance (ANOVA). Statistical analysis was performed using the GraphPad Prism Software (Version 8.0.1, California, United States). A value of *p* < 0.05 was considered statistically significant, and a value of *p* < 0.01 was considered highly significant.

## Results

### Effect of RUT on Gross Evaluation of the Gastric Mucasa in Mice

**FIGURE 2:**
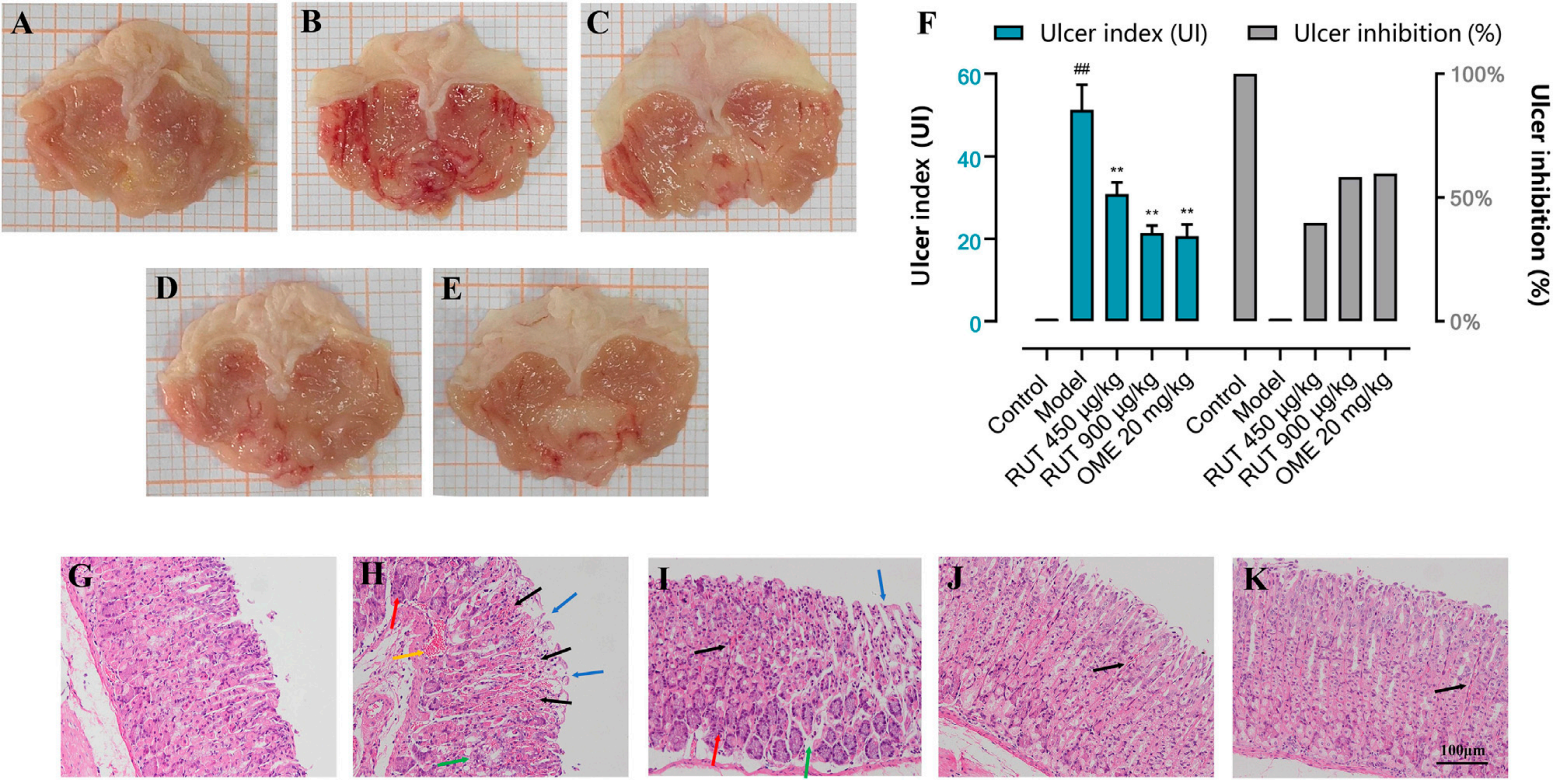
Effect of RUT on ethanol-induced gastric mucosa injury in mice in different groups. Note: **(A–E)** Macroscopic representative images; **(F)** Ulcer index and ulcer inhibition; **(G–K)** Microscopic representative images (H&E stained, ×200 magnification; Scare bar: 100 μm. Blue →: loss of gastric epithelial cells; Black →: hemorrhagic injury; Yellow →: vascular congestion; Green →: edema; Red →: inflammatory cells infiltration). **(A,G)** Normal control group; **(B,H)** Model group; **(C,I)** RUT 450 μg/kg group; **(D,J)** RUT 900 μg/kg group; **(E,K)** OME 20 mg/kg group. Values are expressed as mean ± SD (n = 8). #*p* < 0.05 and ##*p* < 0.01 when compared with the control group. *p < 0.05 and **p < 0.01 when compared with the model group.

Acute gastric mucosal injury was induced by intragastric administration of absolute ethanol. Figure 2 shows that no visible lesions developed in the normal control group. The RUT groups or OME group showed extensively reduced gastric injury compared with the model group, which showed severe gastric mucosal damage appearing as glandular area hyperemia, mucosal edema accompanied by dot and linear hemorrhage necrosis.

Then, in order to reflect the effect of RUT on ethanol stimulation, we used the Guth standard to quantitatively assess the gastric lesions and calculate ulcer inhibition rate. As shown in Figure 2F, the model group indicated by an average UI of 51.25 ± 6.09 (*p* < 0.01). However, pre-treatment with RUT at doses of 450 and 900 μg/kg or omeprazole (20 mg/kg) produced a significant reduction in the percentage of UI (by 39.76, 58.28 and 59.75%, respectively) when compared to the model group (*p* < 0.01).

### Effect of Rutaecarpine on Histopathological Assessment of Gastric Damage

In order to evaluate the protective effect of RUT on the stomach in microscopic conditions, we conducted histopathological analysis. Figures 2G–K shows the histopathological alterations in gastric specimens of different experimental groups. The normal control group showed normal histological structures of the mucosa, submucosa as well as the muscularis. Serious damage of gastric mucosa was found in the submucosa of the model group, and the superficial gastric epithelium was disrupted and exfoliated. Moreover, vascular congestion, edema and inflammatory cells infiltration were also observed. It was clear that the gastric mucosa damages were attenuated with omeprazole or RUT demonstrated by the integrity of superficial gastric epithelium and improvement of hemorrhagic injury, edema and inflammatory infiltration Notably, the pretreatment of mice with 900 μg/kg of RUT conferred the most gastroprotection.

### Effect of Rutaecarpine on Gastric NF-κB Activation and Its Downstream Inflammatory Cytokines Levels

**FIGURE 3:**
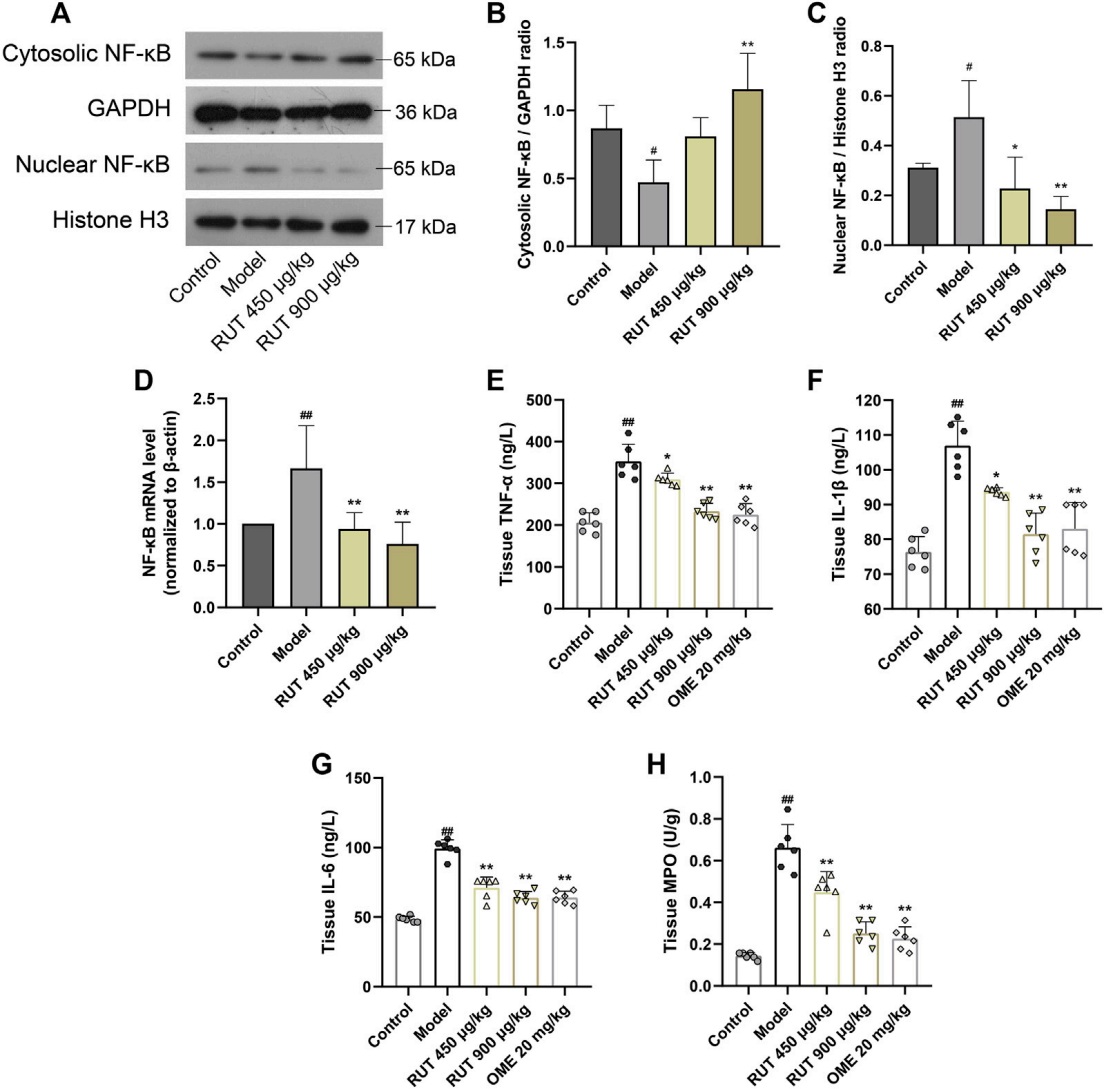
Effect of RUT on inflammatory factors levels in gastric damage induced by ethanol. Note: **(A)** Western blot images of cytosolic and nuclear NF-κB p65; **(B)** Cytosolic NF-κB p65 protein level; **(C)** Nuclear NF-κB p65 protein level; **(D)** NF-κB p65 mRNA level; **(E)** Tissue TNF-α level; **(F)** Tissue IL-1β level; **(G)** Tissue IL-6 level; **(H)** Tissue MPO level. Values are expressed as mean ± SD (n = 6). #*p* < 0.05 and ##*p* < 0.01 when compared with the control group. *p < 0.05 and **p < 0.01 when compared with the model group.

**FIGURE 4:**
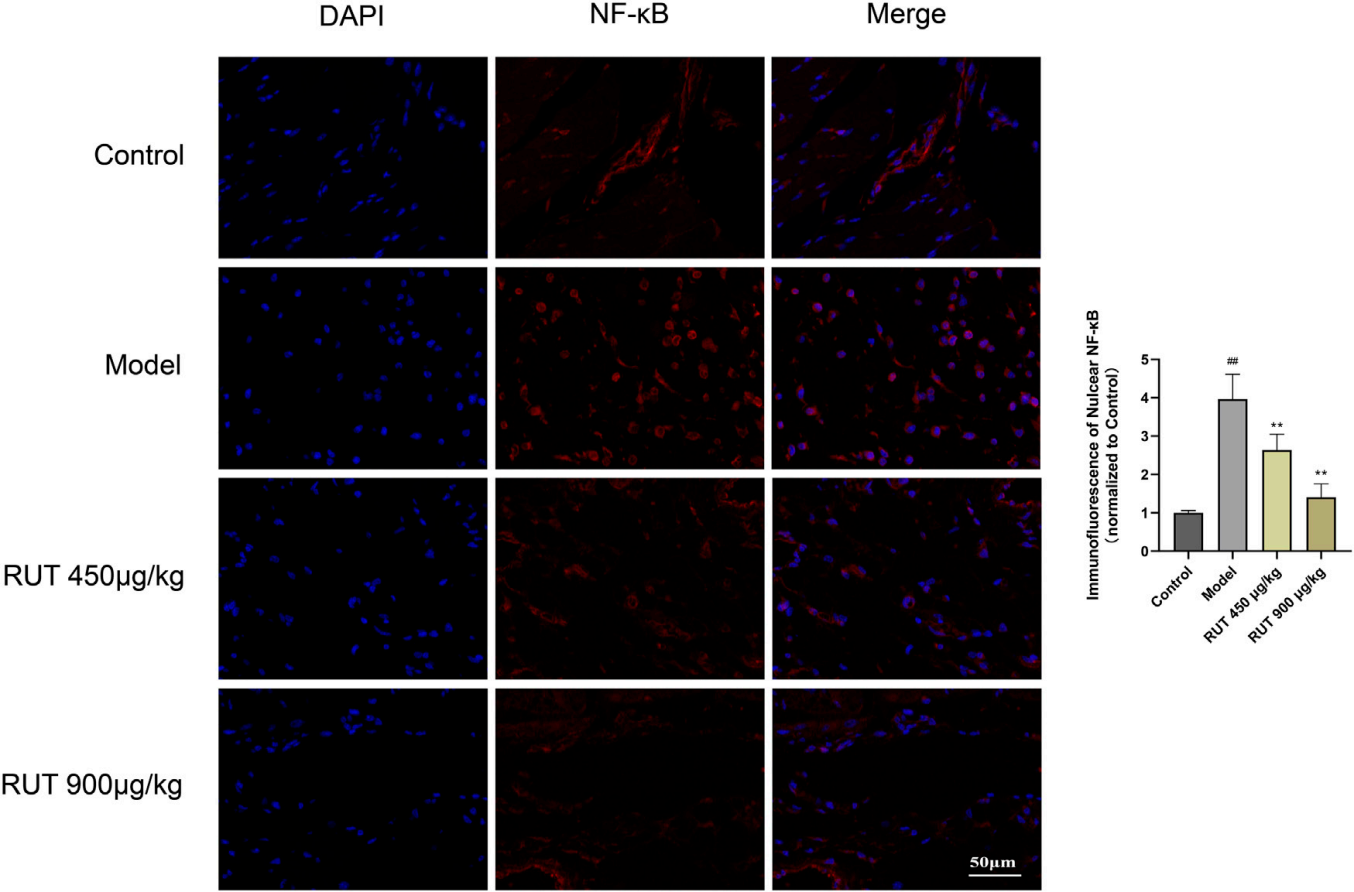
Immunofluorescence analysis for the effect of RUT on NF-κB nuclear translocation (IF ×400 magnification; Scare bar: 50 μm; n = 3).

Next, we assessed the variation of inflammatory factors in gastric mucosa aiming at exploring the intervene of RUT on inflammation. As revealed in Figure 3, the inflammatory disturbances were evaluated by monitoring the NF-κB nuclear translocation and alterations in its downstream signals. In terms of results, ethanol intake leaded to a sharp increase in NF-κB expression and nuclear translocation compared with control group (Figures 3A–D). Meanwhile, it also upregulated the level of some proinflammatory cytokines, such as TNF-α, IL-1β, IL-6 as well as MPO (Figures 3E–H). While performance of RUT pretreatment attenuated the expression and nuclear translocation of NF-κB. In addition, RUT reversed the level of TNF-α, IL-1β, IL-6 and MPO effectively, which is consistent with the results after OME processing. We also additionally used immunofluorescence to analyze the nuclear translocation of NF-κB, and the result also verified the previous results (Figure 4). All of which prove that the anti-inflammatory effect of RUT can relieve gastric mucosal damage.

### Effect of Rutaecarpine on Ethanol-Triggered Oxidative Stress and Antioxidant Enzumes Activity

**FIGURE 5:**
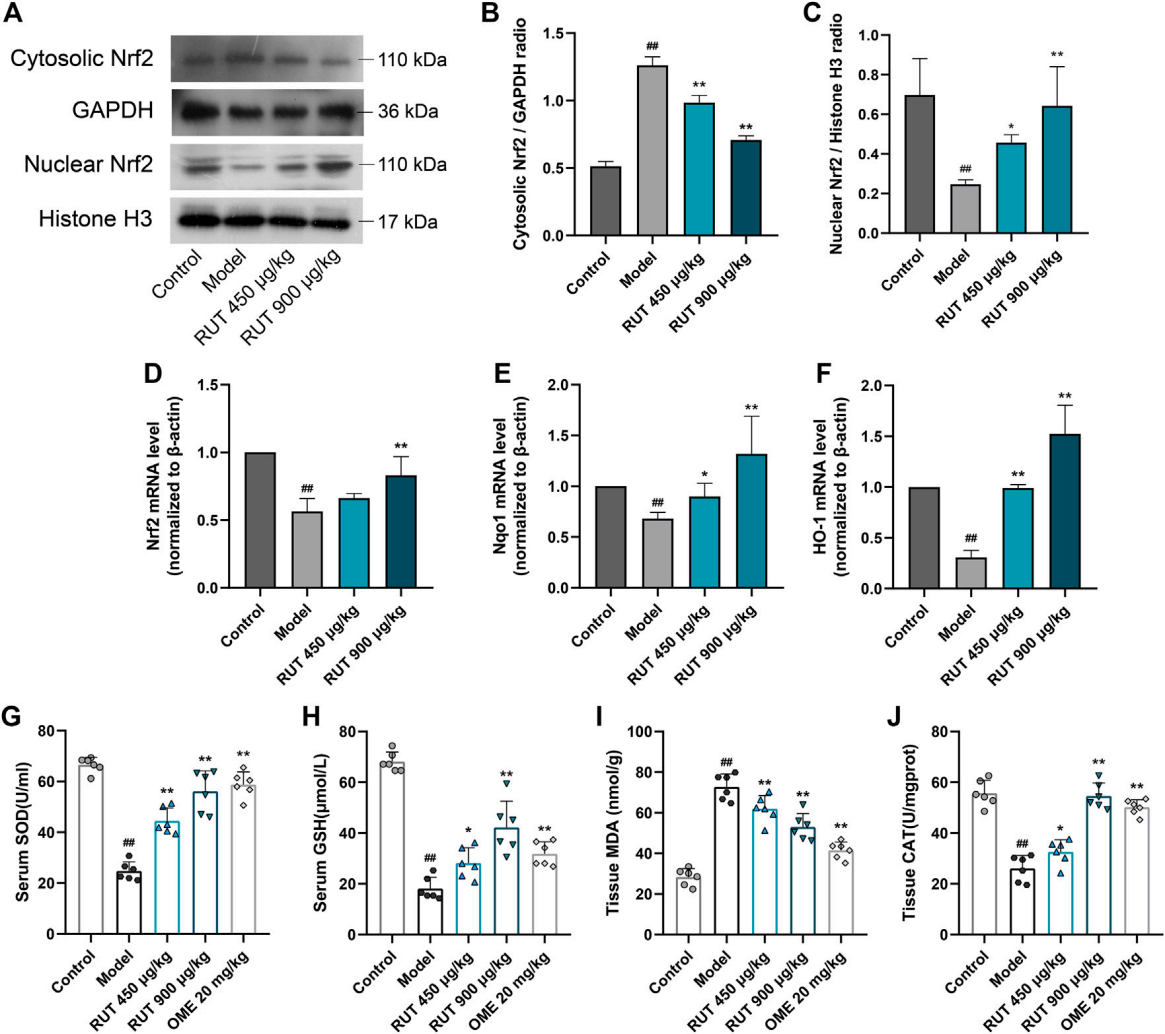
Effect of RUT on oxidative stress factors levels in gastric damage induced by ethanol. Note: **(A)** Western blot images of cytosolic and nuclear Nrf2; **(B)** Cytosolic Nrf2 protein level; **(C)** Nuclear Nrf2 protein level; **(D)** Nrf2 mRNA level; **(E)** Nqo1 mRNA level; **(F)** HO-1 mRNA level; **(G)** Serum SOD level; **(H)** Serum GSH level; **(I)** Tissue MDA level. Values are expressed as mean ± SD (n = 6). #*p* < 0.05 and ##*p* < 0.01 when compared with the control group. *p < 0.05 and **p < 0.01 when compared with the model group.

Then, we explored the stimulation of oxidative stress in the gastric mucosa by monitoring changes in antioxidants and lipid peroxidation levels. Exposure to ethanol presented a high level of lipid peroxidation, which was indicated by the increase of MDA (Figure 5I). In addition, the intake of ethanol significantly inhibited the activity and nuclear translocation of the antioxidant element Nrf2 (Figures 5A–D), accompanied by a decrease in its downstream signals levels, such as Nqo1, HO-1, SOD, CAT and GSH (Figures 5E–H), of which the levels of SOD, CAT and GSH were the same as those after OME treatment. RUT obviously offsets these oxidation distortions as proved by the reversal of the levels of these factors. Therefore, these results indicated that the involvement of RUT antioxidation activity for the restoration of gastric damages partly.

### Effect of Rutaecarpine on Apoptosis Factors Levels and PI3K/AKT Pathway

**FIGURE 6:**
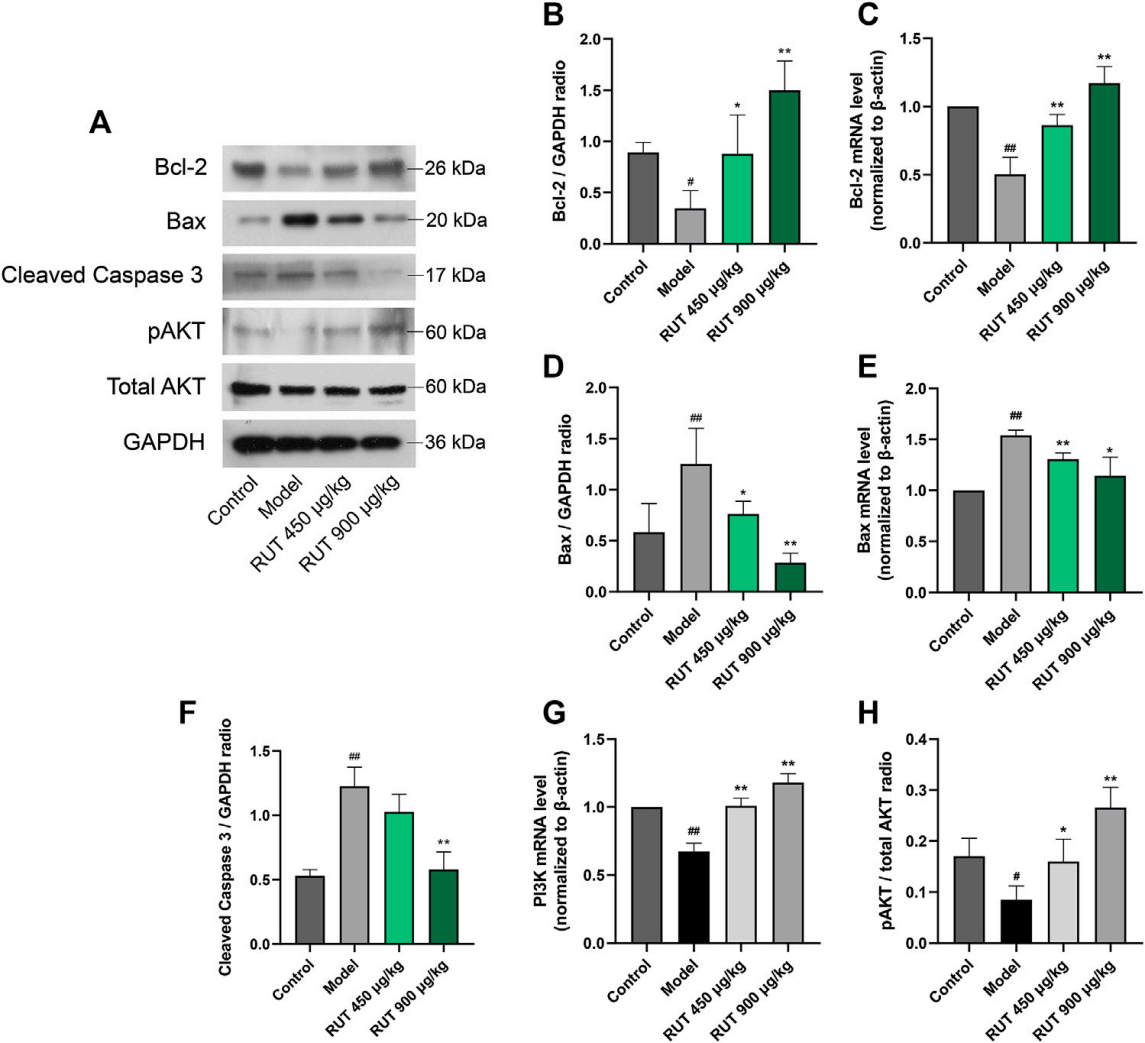
Effect of RUT on apoptosis factors levels and PI3K pathway in gastric damage induced by ethanol. Note: **(A)** Western blot images of Bcl-2, Bax, pAKT and total AKT; **(B)** Bcl-2 protein level; **(C)** Bcl-2 mRNA level; **(D)** Bax protein level; **(E)** Bax mRNA level; **(F)** Tissue Caspase 9 level; **(G)** Caspase 3 mRNA level; **(H)** Tissue Caspase 3 level; **(I)** PI3K mRNA level. **(J)** pAKT protein level; Values are expressed as mean ± SD (n = 6). #*p* < 0.05 and ##*p* < 0.01 when compared with the control group. *p < 0.05 and **p < 0.01 when compared with the model group.

At last, for effect of RUT on apoptosis, we measured the production of apoptotic signals and the activation of the PI3K/AKT pathway. It could be clearly observed that ethanol triggered gastric apoptosis as proved by the pronounced reduction in the level of Bcl-2 (Figures 6A–C) and the increase in the level of Bax (Figures 6A,D,E), and Caspase 3 (Figures 6A,F) when compared with the normal control group. In addition, ethanol inhibited the activity of PI3K/AKT pathway significantly. It is worth noting that RUT counteracted these mutations as demonstrated by the decline of Bax and restoration of Bcl-2. And Caspase 3 maintained its previous level after OME treatment. Besides, RUT restored the mRNA expression of PI3K and protein expression of pAKT. These results demonstrate that RUT plays a considerable role in ameliorating the apoptosis situation in gastric mucosa and activating PI3K/AKT pathway, which is implicated in RUT gastroprotective effects.

## Discussion

GU is a multifactorial gastrointestinal disease, of which alcohol is the biggest contributing factor (Das and Banerjee, 1993). In this previous study, due to the functional and anatomical similarity to the human stomach, the mice were chosen as the model and administered ethanol intragastrically to simulate human GUs caused by excessive drinking. Additionally, in several frequently used models, such as ethanol, pylorus ligation, non-steroidal anti-inflammatory drugs (NSAIDs) and stress-induced GU, the ethanol model is one of the widely used experimental models, which is similar to lots of characteristics of human acute peptic ulcer disease (Deding et al., 2016; Li et al., 2018). However, the mechanism of ethanol damage to gastric mucosa is not completely clear. Previous studies have shown that it is related to the direct damage of gastric epithelial cells (Zhao et al., 2009) and mucus layer (da Silva et al., 2018), or the indirect damage such as the influence of gastric mucosal hemodynamics (Kawano and Tsuji, 2020), infiltration of leukocytes and ensued inflammatory (Li et al., 2017) and oxidative stress (Loguercio et al., 2009; Salaspuro, 2011) and apoptosis distortion (Arab et al., 2019). Thus, this present study focused on the forthputting of RUT to treat ethanol-induced gastric injury through anti-inflammatory, anti-oxidation and anti-apoptosis.

In term of our results, ethanol intake could trigger severe inflammation in stomach, accompanied by activation of the NF-κB pathway and up-regulation of its downstream signal, such as TNF-a, IL-1B, IL-6 and MPO. These findings are in line with previous researches (Raish et al., 2018; Yeo et al., 2018; ). In fact, NF-κB is expressed in almost all cells and performs a nonnegligible role in the pathogenesis of GU. Four transcript variants encoding different isoforms have been found including NF-κB p65/p105/p50/p52. In this article, we detected NF-κB p65 due to its contribution to inflammation. Many pro-inflammatory stimuli and ROS can lead to the activation of NF-κB through the phosphorylation of inhibitors of κB (IκBs) by the IκB kinase (IKK) complex (Akanda et al., 2018; Chen et al., 2001; Arab et al., 2019). Afterwards, free NF-κB translocates into the nucleus and consequently results in the transcriptional activation of a variety of pro-inflammatory mediators, such as TNF-α, IL-1β and IL-6 and MPO (Mitsuyama et al., 2006). Interestingly, RUT mitigated the gastric damage by inhibiting the NF-κB pathway, and then suppressed downstream proinflammatory elements. These correlates were consistent with mitigation of leukocyte infiltration. It is undoubtedly an efficacious strategy for the treatment of GU to ameliorate the aberration of these inflammatory pathological factors. In fact, RUT has pronounced anti-inflammatory capabilities in a variety of disease models according to the reports (Li et al., 2019a; Li et al., 2019b; Luo et al., 2020), which also corroborates our results from the side.

It is familiar that in addition to inflammation, the excessive generation of ROS also affects oxidative stress, which leads to altering the endogenous antioxidant defense system, including SOD and GSH (Arunachalam et al., 2019). The main source of ROS in ethanol-damaged gastric tissue is infiltration of activated neutrophils, which MPO is a crucial indicator of neutrophil infiltration in ulcer-induced injuries (Arab et al., 2015; Paulrayer et al., 2017). Lipid peroxidation is consequence of ROS reaction against cell membrane and produces significant levels of MDA, which leads to oxidative gastric damage (Yu et al., 2017). The present findings indicated that the application of ethanol indulged MDA and significantly inhibited the production of the Nrf2. These changes prevented transcription factor Nrf2 from serving as a sensor to regulate the expression of antioxidant enzymes (HO-1 and Nqo1) driven by antioxidant response elements (ARE) in response to oxidative stimulation. The observations were consistent with previous researches (Nguyen et al., 2009; Itoh et al., 2010). Interestingly, our results show that RUT remarkably reverses the redox induced by ethanol. A good explanation for inhibiting oxidative stress was the observed remission of neutrophil infiltration and the decrease of membrane lipid peroxidation level. Ample evidence demonstrated the antioxidants plays a central role in process of GU (Kwiecien et al., 2002; Raish et al., 2018; Mohan, et al., 2020). Our data validated the restoration of Nrf2 along with the prototypical Nrf2 target genes (HO-1 and Nqo1) and the replenish of crucial antioxidation elements (SOD, CAT and GSH) in the wake of RUT treatment. All of data suggest the potential prospects of RUT in treating GU through anti-oxidation.

Numerous studies demonstrated that apoptosis also goes hand in hand with the occurrence of GU, and the continuous excessive production of apoptosis will destroy the integrity of gastric mucosa and eventually induce gastric mucosa dysfunction (Liu et al., 2020). Mechanistically, our data indicated ethanol intake made the anti-apoptosis gene Bcl-2 down-regulated markedly driven by ROS and pro-inflammatory signals. In this circumstance, the restriction to the apoptotic protein Bax was limited, which in turn leaded to the mitochondrial escape of cytochrome C and subsequently activated Caspase 9 and Caspase 3. This is consistent with previous studies (Raish et al., 2018; Xie et al., 2020). After receiving RUT, it could be clearly observed that the anti-apoptosis capacity of gastric mucosa was enhanced, manifested in the recovery of BCL-2 level and the control of Bax and Caspase 3. Actually, previous studies have demonstrated the latent anti-apoptosis activity (Li et al., 2019a; Li et al., 2019b; Han et al., 2019). And our data strongly indicated that RUT could partly participate in the palliation of the damage caused by ethanol though anti-apoptosis.

The present findings also revealed that ethanol inhibited PI3K/AKT pathway, which is consistent with previous literature (Zhang et al., 2019). In fact, the activation of PI3K/AKT pathway is essential for cell resistance to oxidation and apoptosis (Arab et al., 2019; Cao et al., 2020). Our data suggest that the activation of AKT phosphorylation promoted by RUT was associated with the Nrf2 and Bcl-2 up-regulation and in turn to the down-regulation of Bax.

**FIGURE 7:**
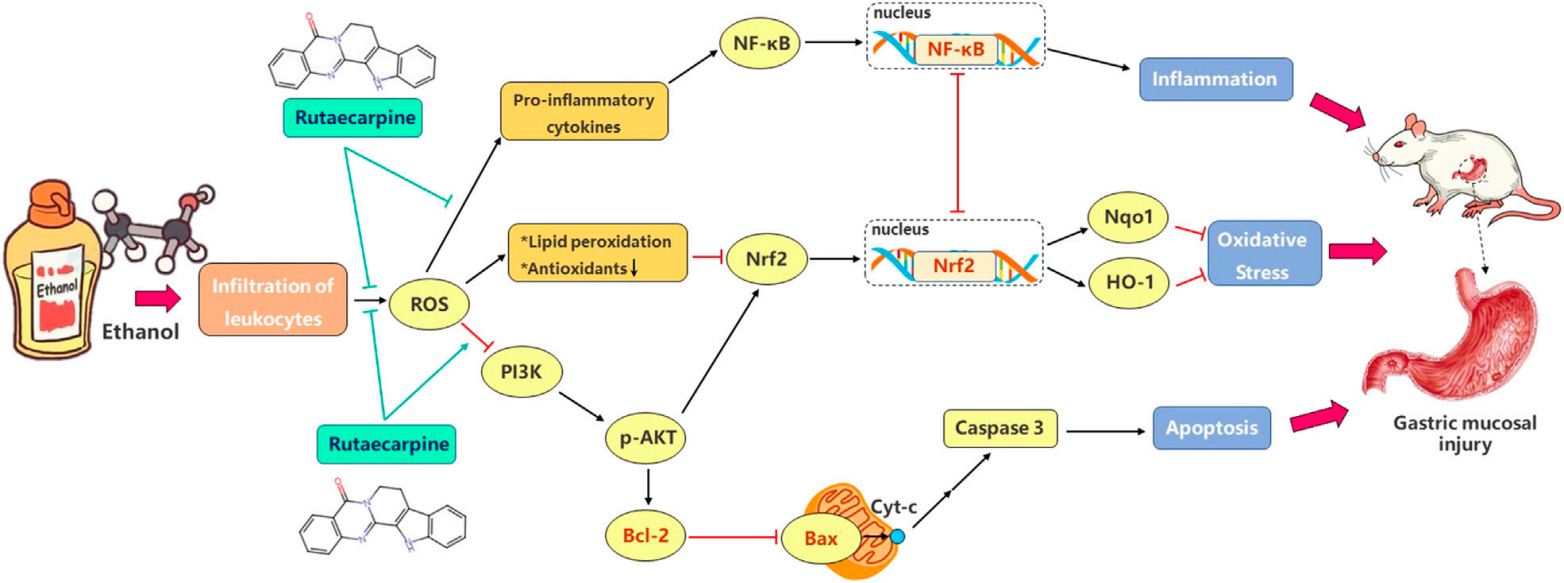
Schematic diagram of signal pathway that mediates ethanol-induced gastric mucosal injury and the ameliorative effects of RUT on mice. (→: activate; ˧: inhibit).

In summary, inflammation, oxidative stress and apoptosis are closely related to the occurrence of GU. The excessive production of ROS released inflammation and apoptosis signals, and inhibited antioxidant elements to promote the continuous production of oxidative stress. Our findings clearly revealed that RUT augments cellular anti-inflammation, antioxidant and anti-apoptosis defense capacities, thereby protecting cells from ethanol damage. The potential mechanism seems to be related to the induction of antioxidant and anti-apoptosis enzymes synthesis by activating Nrf2 and Bcl-2 through PI3K/AKT-dependent pathway, and to the inhibition of NFKB pathway and inflammation signals. Figure 7 showed their complex relationship. These findings indicate that RUT might be a potential therapeutic agent for GU.

## Data Availability Statement

The original contributions presented in the study are included in the article/supplementary material, further inquiries can be directed to the corresponding author.

## Author Contributions

SR and YZ conceived the project and wrote the manuscript. SR performed main part of the experiments, with contributions from YW, RW, SW, and JW. TY, XC, SW, MJ, HL, and MW contributed to the data collection and analysis. YZ participated in the project design as well as manuscript draft preparation and revision. All authors read and approved the final manuscript.

## Conflict of Interest

The authors declare that the research was conducted in the absence of any commercial or financial relationships that could be construed as a potential conflict of interest.

## Glossary

GU: gastric ulcer
RUT: rutaecarpine
OME: omeprazole
UI: ulcer index
NF-κB: nuclear factor κB
TNF-α: tumor necrosis factor-α
IL-1β: interleukin-1β
IL-6: interleukin-6
MPO: myeloperoxidase
DAPI: 4,6-diamidino-2-phenylindole
Nrf2: nuclear factor erythroid 2-related factor 2
Nqo1: NAD(P)H/quinone oxidoreductase 1
HO-1: heme oxygenase-1
SOD: superoxide dismutase
GSH: glutathione
CAT: catalase
MDA: malondialdehyde
Bcl-2: B cell lymphoma-2 expression
Bax: Bcl-2 associated x
Cyt C: cytochrome C
pAKT: phospho protein kinase B
PI3K: phosphatidylinositol 3-kinase
